# Weight loss-induced improvement of body weight and insulin sensitivity is not amplified by a subsequent 12-month weight maintenance intervention but is predicted by adaption of adipose atrial natriuretic peptide system: 48-month results of a randomized controlled trial

**DOI:** 10.1186/s12916-022-02435-9

**Published:** 2022-07-28

**Authors:** Linna Li, Dominik Soll, Verena Leupelt, Joachim Spranger, Knut Mai

**Affiliations:** 1 Department of Endocrinology and Metabolism, Charité – Universitätsmedizin Berlin, corporate member of Freie Universität Berlin, Humboldt-Universität zu Berlin, and Berlin Institute of Health, 10117 Berlin, Germany; 2 Charité-Center for Cardiovascular Research (CCR), Berlin, Germany; 3 DZHK (German Centre for Cardiovascular Research), partner site Berlin, Berlin, Germany

**Keywords:** Insulin sensitivity, Obesity, Weight maintenance, ANP signaling, Quality of life

## Abstract

**Background:**

Behavioral weight loss interventions are frequently hampered by long-term inefficacy. As metabolic improvements and health-related quality of life (HRQoL) are diminished by weight regain, effective long-term strategies are highly desirable. We aimed to analyze whether an additional weight maintenance intervention could delay body weight regain and can induce a long-term improvement of metabolism and HRQoL for up to 48 months in humans. Given the short-term metabolic effects of natriuretic peptides (NP), we also investigated the role of the adipose atrial NP (ANP) system in this long-term context.

**Methods:**

After a successful 12-week weight reduction program 143 subjects (age>18; BMI≥27 kg/m^2^) were randomized (1:1) to a control group or a 12-month multimodal weight maintenance intervention focusing on nutritional counseling and physical exercises. Secondary trial outcomes including course of BMI, HOMA-IR, glucose response after oGTT (glucose_AUC_), and HRQoL (SF-36) were analyzed yearly for 48 months. Adipose ANP receptor mRNA expression was analyzed during weight loss.

**Results:**

Initial weight loss (− 4.7±1.5 kg/m^2^) improved glucose_AUC_, HOMA-IR, and HRQoL. Although BMI was still reduced after 48 months (−1.98 [95% CI −2.61, −1.35] kg/m^2^), benefits on HOMA-IR, glucose_AUC_, and mental health disappeared after 36 (−0.49 [−1.00, 0.02]), 18 (0.61 [−9.57, 10.79] mg dl^−1^ min^−1^), and 18 months (2.06 [−0.08, 4.20]), respectively, while improved physical health persisted up to months 48 (2.95 [0.49, 5.40]). Weight maintenance intervention inhibited weight regain and delayed impairment of HOMA-IR and glucose_AUC_ (but not HRQoL) for up to 12 months. However, no metabolic long-term effect was seen beyond the intervention period. Lower adipose *NPR-C* and higher *NPR-A* mRNA expression after weight loss predicted smaller regain of weight (*r*=0.398; *p*<0.05)/fat mass (FM) (*r*=0.391; *p*<0.05) and longer improvement of HOMA-IR (*r*=−0.422; *p*<0.05), respectively.

**Conclusions:**

Additional benefits of a behavioral 12-month weight maintenance intervention after weight loss regarding body weight regain and metabolic improvement does not persist beyond the intervention period. However, weight loss-induced modulation of the adipose ANP system is probably involved in the long-term control of body weight regain and insulin sensitivity.

**Trial registration:**

ClinicalTrials.govNCT00850629. Registered on February 25, 2009.

**Supplementary Information:**

The online version contains supplementary material available at 10.1186/s12916-022-02435-9.

## Background

Diabetes is one of the major current health issues. It is known to be associated with increased cardiovascular risk [1], high depression rate, and impaired health-related quality of life (HRQoL) [2]. Unfavorable body composition and increased hepatic, muscular, and adipose insulin resistance are crucial components underlying the development of type 2 diabetes. A beneficial effect of weight loss on insulin sensitivity and diabetes prevalence is well established, in general [3–5]. However, despite numerous efforts, sustained weight loss is currently one of the major challenges in the treatment of obesity. Several lifestyle-based intervention trials indicate that weight regain is frequently observed after several months in the majority of individuals with obesity [6–9]. This is usually accompanied by improvement or even remission of numerous metabolic and health benefits achieved by weight loss [3, 10]. Thus, successful strategies to maintain body weight reduction and to realize a long-term improvement of metabolism and HRQoL are urgently required.

A small and comparable reduction of weight regain was demonstrated up to five years by continuous long-term intervention using different approaches including both personal contacts and interactive online support. Nevertheless, substantial weight regain was observed in all groups [7, 8]. Recently, we demonstrated a successful weight maintenance by a 12-month weight maintenance intervention, although it was substantially attenuated 6 months after finishing this intervention [11, 12]. This finding was primarily driven by a stronger weight regain in the intervention group after ceasing the treatment compared to the continuous and constant weight regain in the control group. The long-term course of this dynamic as well as the short- and long-term consequences of such an approach regarding metabolic state and HRQoL have not been analyzed so far. Given this dynamic and the progressive albeit slow natural increase of body weight described after intended weight loss [6], long-term metabolic and health-related benefits of temporary weight-maintaining strategies are of high interest. Therefore, we aimed to investigate the long-term weight trajectory after such a weight loss-weight maintenance intervention and whether this approach can prevent short- and long-term worsening of weight loss-induced improvement of metabolism and HRQoL. Such a legacy effect has been exemplarily observed for microvascular changes after remission of type 2 diabetes after bariatric surgery even if a relapse of type 2 diabetes occurred [13].

Although molecular mechanisms driving weight regain and metabolic improvement are currently not fully understood, it is widely accepted that hormonal circuits may substantially contribute to body weight regulation. Recently we were able to demonstrate the eminent role of the local atrial natriuretic peptide (ANP) system on the regulation of fat mass and insulin sensitivity during acute weight loss [14]. Especially the balance between natriuretic receptor A (NPR-A), which activates a cyclic guanosine monophosphate (cGMP) driven signal cascade resulting in stimulated lipolysis in adipose tissue and mitochondrial biogenesis in skeletal muscle [15], and adipose NPR-C involved in clearing and degradation of circulating ANP might be crucial. The impact of this system on long-term body weight regain and metabolic changes has currently not been investigated. Therefore, we also aimed to explore the role of the adipose ANP system on long-term modulation of body weight and glucose metabolism after weight loss.

## Methods

### Participants

Participants were recruited via newspaper ads or via an endocrine outpatient clinic. A total of 223 subjects (BMI ≥ 27 kg/m^2^) were screened for participation. Subjects with abnormal thyroid function, hypercortisolism (excluded by 1 mg dexamethasone suppression test), severe chronic diseases, such as unstable coronary heart disease, severe renal insufficiency (eGFR< 30 ml/min), liver diseases, severe psychological diseases, severe endocrine disorders, cancer, chronic infections or comparable chronic disorders were excluded. Moreover, individuals with changes of smoking habits or dietary behavior during the last 3 months, and recent weight changes of more than 5 kg during the last 2 months, were also excluded. Drugs modifying energy homeostasis and body weight were not allowed during this trial (with exception of thyroxin). The study protocol was approved by the Institutional Review Board of the Charité Medical School and all subjects gave written informed consent prior to inclusion in the study. The trial was registered at ClinicalTrials.gov (NCT00850629).

### Study design

Details of the performed weight loss-weight maintenance trial in adults (Maintain-Adult) have been described previously [11, 14]. The major characteristics of the trial are shown in figure S1. In summary, after an initial weight loss period, we performed a randomized controlled trial which compared the effect of a 12-month multimodal lifestyle intervention to maintain body weight with a control group. The study was performed between 2010 and 2016 at a University Center.

#### Pre-trial weight loss phase

In brief, 143 of 156 Caucasians with overweight or obesity (120 female and 36 male; 50.5 [41.7, 60.8] years) successfully underwent a 12-week multimodal structured weight reduction program (weight loss of at least 8%). This is based on caloric restriction using a very low energy diet (Optifast 2®, 800 kcal, Nestlé HealthCare Nutrition GmbH, Frankfurt am Main, Germany), nutritional counseling, and increased physical activity. The detailed protocol has been reported previously [14] and is given in the Additional file 1: Appendix S1.

#### Twelve-month randomized weight maintenance phase

Subjects who lost at least 8% of their body weight during the weight loss phase were considered to be eligible for randomization (*n*=143, 112 female and 31 male). They were randomized into the intervention or the control group. Whereas subjects in the control group were no longer involved in any form of counseling, a continuous counseling program including 36 meetings was performed for the next 12 months in the intervention group in a gradually decreasing frequency. These were comparable to sessions of the weight loss period. The dietary advice was focused on a balanced diet with a comparable distribution of macronutrients as recommended within the final phase of the weight loss intervention. An individual caloric intake was calculated and adapted to achieve body weight maintenance, even if further weight loss was allowed. A decreased energy intake (500 kcal below the calculated energy demand) was recommended in case of weight gain. The supervised physical activity regime was maintained for the first 12 weeks of the weight maintenance period. Afterwards, individuals were encouraged to exercise at least twice a week without direct supervision. Pedometers were given to the participants and a gym membership was offered. The psychological support was continued for six additional dates. Details of the protocol and the intervention have been reported previously [11, 14] and are extensively described in the Additional file 1: Appendix S1.

#### Follow-up period

After 12 months all subjects (intervention and control group) underwent a free living period of 36 months without any further active intervention up to month 48.

### Randomization and masking

Randomization was performed by the principal investigator using a stratified randomization list. Stratification considered gender and body weight at baseline (three BMI strata). Study nurses and physicians but not participants were blinded to group assignment. This includes all phenotyping procedures, assessment of primary and secondary outcomes as well as data analysis.

### Phenotyping

A comprehensive phenotyping was performed before (T−3) and after (T0) weight loss, 12 months (T12) after randomization, and during follow-up until month 48 (T18, T24, T36, and T48). This included anthropometric, hormonal, and metabolic evaluation using oral glucose tolerance test, bioimpedance analysis (to assess fat mass (FM) percentage) as well as assessment of HRQoL at every time point. Moreover, 24-h urine collection and adipose tissue biopsies were performed before and after weight loss. To avoid interactions between the study procedures, the phenotyping procedures were planned and carried out at intervals of at least 2 days. Details of the phenotyping protocol have been reported [11, 12, 14] and all procedures are extensively described in the Additional file 1: Appendix S1.

### Laboratory tests

Laboratory analyses including measurement of capillary blood glucose, potassium, sodium, calcium, phosphorus, iron, ferritin, serum creatinine, triglycerides, cholesterol, LDL- and HDL-cholesterol, protein, CRP and urea, uric acid, liver enzymes, leucocytes, erythrocytes, thrombocytes, hemoglobin, insulin, and urinary metanephrine levels were performed using established methods. Details including inter- and intra-assay coefficients of variance (CVs) are provided in Additional file 1: Appendix S1. Tissue samples were analyzed by RNA sequencing using the HiSeq2000 system (TruSeq SBS Kit-Hs 200 cycles, Illumina San Diego, US) (details see Additional file 1: Appendix S1).

### Primary and secondary outcomes

The primary outcome was weight regain after 18 months and was defined as an absolute change of BMI from T0 to T18 (kg/m^2^) [11]. Here, we reported secondary outcomes including the trajectory of BMI, insulin resistance and glucose metabolism over 48 months as well as the prediction of body weight and FM regain and insulin sensitivity by estimates of adipose ANP system in Maintain-Adult. Whole-body insulin sensitivity was assessed by HOMA-IR [16]. HRQoL was assessed by Short Form 36 (SF-36) questionnaire [17].

### Power calculation

Weight change up to T18 was used for power calculation as reported previously [11]. Using an α- and β-error rate of 5 and 20% the power calculation resulted in 46 individuals per treatment arm (query 7.0). A total of at least 144 individuals had to be included in the weight reduction period (T−3) as we calculated a 20% dropout rate during the initial weight loss period and about 15% dropouts during the randomized intervention.

### Statistics

Statistical procedures were performed using SPSS version 22.0 (SPSS Inc., Chicago, IL, USA) and SAS software, version 9.4 (SAS Institute). Comparison between baseline and after weight loss was done via paired Student’s t-test for normally distributed data and Wilcoxon test for skewed data. Correlations between variables were assessed by the Spearman coefficient of correlation. Weight loss-induced changes of each parameter were expressed as absolute changes compared to baseline (T−3). We calculated the area under the curve (AUC) using the trapezoidal method for analysis of glucose response after oral glucose load (75 g). Disturbances of glucose metabolism were defined based on capillary blood glucose at fasting state and during oral glucose load (normal: capillary fasting glucose (0-G) < 90 mg/dl and capillary blood glucose 120 min after glucose intake (120-G) < 140 mg/dl; impaired fasting glucose: 0-G ≥ 90 mg/dl and < 110 mg/dl and 120-G < 140 mg/dl; impaired glucose tolerance: 0-G < 90 mg/dl and 120-G ≥ 140 mg/dl and < 200 mg/dl; diabetes: 0-G ≥ 110 mg/dl or 120-G ≥ 200 mg/dl) as well as intake of antidiabetic drugs. Frequency of disturbances of glucose metabolism was compared by pairwise McNemar’s test.

Time course of the analyzed outcome parameters and their changes compared to baseline were analyzed by a mixed-model, repeated-measures analysis of variance, which considered the correlation between repeated observations and used all available subsequent observations for all participants with values at randomization, regardless of further assessment completion. Means were modeled as a function of group assignment and study visit (T0, T12, T18, T24, T36, and T48). The models included adjustment for the treatment group, sex, age, BMI at baseline, time and interaction of time, and randomization state as fixed effects. An unstructured covariance structure was used. Data were presented as means and 95%CI. P values were adjusted for multiple testing using Bonferroni correction. We performed a per-protocol analysis. Only the analysis of the BMI was additionally performed on an intention-to-treat basis using a model comparable to per-protocol analysis. Therefore, missing data of the randomized participants were imputed by the rather conservative last observation carried forward (LOCF) method to avoid effect overestimation.

To analyze the independent effect of estimates of the ANP system after weight loss at T0 on ΔBMI_T0T48_, ΔFM_T0T48_, ΔHOMA-IR_T0T48_, and ΔAUC_glucoseT0T48_, we performed linear regression analyses, including age, sex, treatment group and BMI (or FM, HOMA-IR or AUC_glucose_, respectively) after weight loss (at time of randomization (T0)) as covariates.

Results were considered to be significant if the two-sided α was below 0.05. Data are presented as median and limits of the interquartile range (IQR: 25th–75th percentile), as mean with standard deviation, or as group sizes and proportions if appropriate. Estimates of effects are presented as means and 95% confidence intervals unless otherwise mentioned.

## Results

### Body mass index

A flow chart of the trial is shown in Additional file 1: Fig. S1. The results of the weight loss period have been reported previously [14] and are presented in Additional file 1: Table S1. In summary, the weight loss intervention reduced BMI of the entire cohort by 4.7±1.5 kg/m^2^, which was associated with improvement of numerous metabolic parameters. Baseline characteristics after weight loss did not substantially differ between intervention and control subjects, even if triacylglycerol levels were slightly lower in the intervention group (Table 1).

**Table 1:** Metabolic and anthropometric parameters of the randomized participants after weight loss. Results were presented as median and IQR or mean±SD, and *n* represents the number of participants

Parameter	*n*	Intervention group		*n*	Control group		*p* value
Age [year]	72	50.3 [41.6, 60.4]		71	51.3 [43.8, 61.4]		0.195
BMI [kg/m^2^]	72	31.2 [28.5, 35.3]		71	31.3 [28.8, 37.4]		0.462
Use of diuretics [n]		8			7		
Waist circumference [cm]	72	96.5 [88.1, 105.9]		71	99.0 [88.0, 110.0]		0.292
Total cholesterol [mg/dl]	72	175.7±36.7		71	171.9±32.7		0.513
HDL-cholesterol [mg/dl]	72	46.6 [39.2, 56.1]		71	47.4 [39.0, 57.5]		0.905
LDL-cholesterol [mg/dl]	71	109.2±31.1		71	103.3±29.6		0.244
Triacylglycerol [mg/dl]	72	77.0 [59.5, 105.0]		71	88.7 [68.4, 128.0]		0.020
ISI_Clamp_ [mg kg^−1^ min^−1^/[mU l^−1^]]	71	0.08 [0.07, 0.11]		69	0.08 [0.06, 0.10]		0.587
Systolic blood pressure [mmHg]	71	119.0 [113.0, 126.3]		71	119.0 [110.0, 126.7]		0.587
Diastolic blood pressure [mmHg]	71	74.3 [69.0, 81.7]		71	71.7 [68.0, 79.3]		0.242
Adipose NPR-A mRNA expression	44	4785 [4328, 5327]		31	4786 [4393, 5540]		0.889
Adipose NPR-C mRNA expression	44	456 [279, 629]		31	528 [279, 1004]		0.241

*BMI* body mass index, *HDL-cholesterol* high-density lipoprotein cholesterol, *LDL-cholesterol* low-density lipoprotein cholesterol, *ISI*_*Clamp*_ Insulin sensitivity index assessed by hyperinsulinemic euglycemic clamp, *NPR* natriuretic receptor

**Fig. 1:**
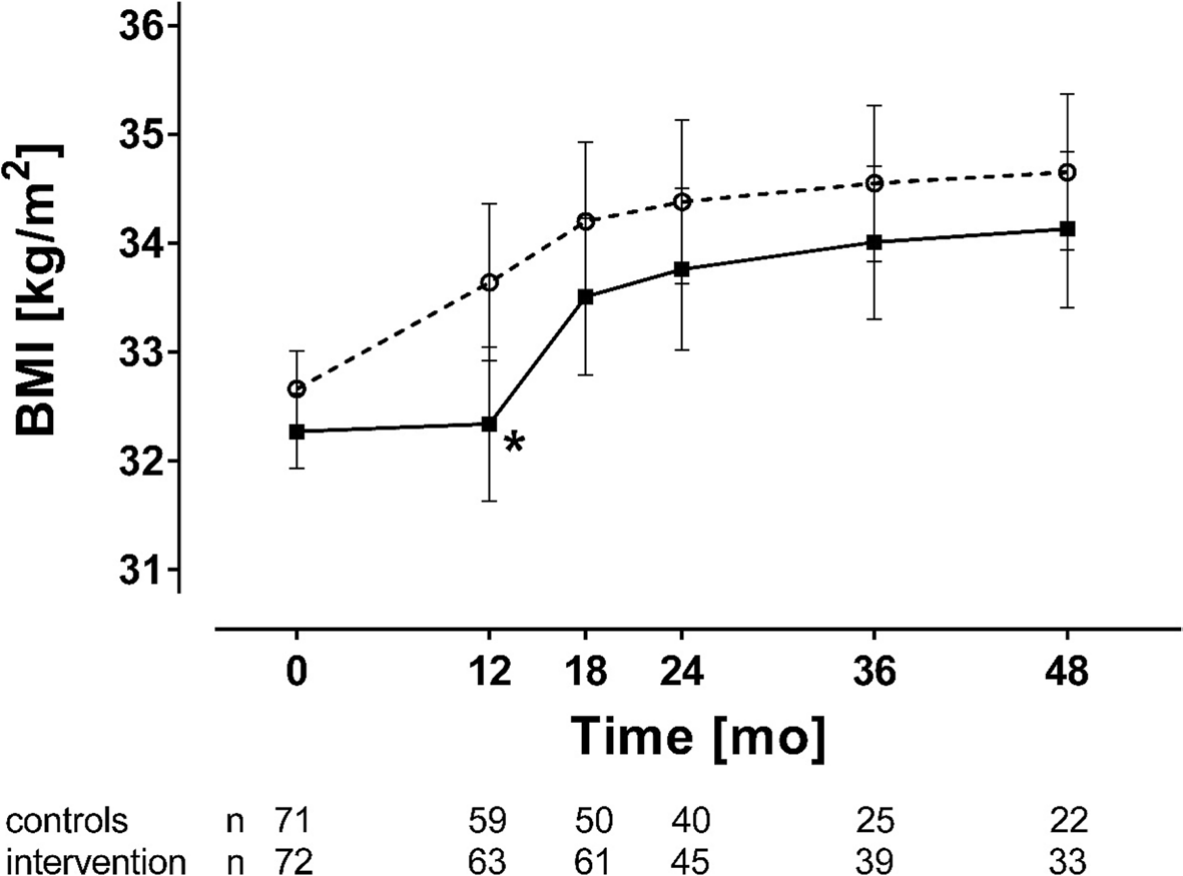
BMI during the randomized controlled trial. Results of the intention to treat analysis of BMI of the control (open circles) and intervention group (filled squares) during the maintenance period (month 0 to month 12) and follow-up (up to 48 months). Results were presented as mean ± 95%CI. *p < 0.05 control vs. intervention group, for comparison of least square means adjusted for age, sex, and BMI after weight loss

Although a substantial weight regain of 2.82 [95% CI; 2.06, 3.58] kg/m^2^ (adjusted for sex, age, and BMI at baseline) occurred until month 48 (T48), BMI was still reduced at T48 compared to baseline (−1.98 [−2.61, −1.35] kg/m^2^) (adjusted for sex, age, and BMI at baseline). As previously reported, the multimodal lifestyle intervention over 12 months prevented BMI regain within the first 12 months [11]. However, there was apparently no long-term effect on BMI course beyond the intervention period (Table 2). Intention to treat analysis resulted in similar results (Fig. 1 and Additional file 1: Table S2).

**Table 2:** BMI, HOMA-IR, and AUC_glucose_ [mean and 95% CI] and estimated mean differences within the per-protocol analysis. Intervention effects are reported as estimated marginal means and estimated mean differences [intervention minus control] based on a mixed-model, repeated-measures analysis of variance adjusted for the treatment group, sex, age, and BMI at baseline

Months after weight loss	Intervention group [kg/m^2^]	Control group [kg/m^2^]	Group difference
BMI [kg/m^2^]			
0	31.46 [31.11, 31.81]	31.84 [31.48, 32.20]	-0.39 [-0.84, 0.07]
12	31.71 [31.00, 32.44]**	33.05 [32.29, 33.82]	-1.35 [-2.39, -0.31]
18	33.04 [32.30, 33.79]	33.79 [33.01, 34.56]	-0.74 [-1.80, 0.31]
24	33.47 [32.69, 34.25]	34.09 [33.28, 34.91]	-0.63 [-1.74, 0.48]
36	33.90 [33.15, 34.65]	34.42 [33.60, 35.25]	-0.52 [-1.61, 0.57]
48	34.21 [33.39, 35.03]	34.72 [33.78, 35.66]	-0.51 [-1.74, 0.72]
HOMA-IR			
0	1.44 [1.22, 1.67]	1.68 [1.44, 1.91]	-0.24 [-0.53, 0.06]
12	1.60 [1.32, 1.87]*	2.07 [1.77, 2.37]	-0.47 [-0.86, -0.09]
18	2.05 [1.67, 2.42]	2.43 [2.03, 2.84]	-0.39 [-0.93, 0.16]
24	1.98 [1.62, 2.35]	2.36 [1.97, 2.75]	-0.37 [-0.90, 0.15]
36	2.57 [2.01, 3.13]	2.77 [2.11, 3.43]	-0.20 [-1.05, 0.65]
48	2.52 [1.91, 3.14]	3.45 [2.71, 4.19]	-0.93 [-1.88, 0.03]
AUC_glucose_ [mg dl^−1^ min^−1^]			
0	270 [256, 285]	271 [256, 286]	-0.92 [-20.10, 18.26]
12	275 [257, 292]*	300 [282 –318]	-25.65 [-49.32, -1.99]
18	293 [277, 309]	305 [289, 322]	-12.65 [-34.36, 9.05]
24	297 [277, 318]	325 [304, 347]	-27.77 [-56.09, 0.56]
36	315 [294, 335]	343 [320, 365]	-27.68 [-56.54, 1.18]
48	327 [305, 348]	344 [320, 368]	-17.85 [-48.78, 13.08]

*BMI* body mass index, *HOMA-IR* Homeostatic Model Assessment for insulin resistance, *glucoseAUC* area under the curve of glucose response after oGTT

^*^p<0.05

^**^p=0.01 vs. control group

### Modulation of insulin sensitivity and glucose metabolism

Insulin resistance, assessed by HOMA-IR, was substantially improved in the entire cohort by weight loss (−1.60 [−1.99, −1.21] (adjusted for sex, age, and BMI at baseline), resulting in a higher number of subjects with normal glucose metabolism, even if this difference marginally failed to be significant (*p*=0.071) (Additional file 1: Table S3). In line with the continuous increase of BMI after the end of the weight loss period, there was a subsequent increase of HOMA-IR (+1.46 [0.94, 2.08]) in the entire study population until T48. Although BMI was still reduced after 36 and 48 months compared to baseline, HOMA-IR was no longer different from baseline at 36 (−0.49 [−1.00, 0.02] and at 48 months (−0.14 [−0.64, 0.36] (adjusted for sex, age, treatment group, and BMI at baseline) (Additional file 1: Table S4). Like BMI, insulin resistance remained reduced in the intervention group up to the end of the intervention, while a continuous increase occurred within the control group. However, the effect on insulin resistance could not be preserved beyond the end of the maintenance intervention (Table 2).

Glucose levels after oral glucose intake (glucose_AUC_) were not different between both groups at baseline (intervention 277 [244, 318] vs. controls 275 [250, 314] mg dl^−1^ min^−1^; *p*=0.610) and were improved by weight loss (Additional file 1: Table S1). However, unlike HOMA-IR, the continuous increase of glucose_AUC_ after weight loss already resulted in higher levels by 24 months in comparison to baseline (T−3) (Additional file 1: Table S4). Although between-group analysis revealed lower glucose_AUC_ levels after 12 months in the intervention group, no prolonged effect of the maintenance intervention could be observed beyond the end of the intervention period (Table 2).

### Health-related quality of life

All physical and mental components of the HRQoL assessment (SF-36) were substantially increased by weight loss (Additional file 1: Table S5). This resulted in an improvement of both, the physical component summary score (PCS; 46.0 [36.0, 53.4] vs. 51.7 [45.1, 55.8], *p*=7.8×10^−9^) and the mental component summary score (MCS; 49.6 [39.4, 54.8] vs. 53.4 [47.9, 57.3], *p*=7.4×10^−5^) during weight loss. However, only the increase of PCS was significantly associated with the decrease of BMI (*r*=-0.321; *p*=0.32×10^−3^). In contrast to MCS, the improvement of PCS remained significant up to 48 months after weight loss (Table 3). The 12-month maintenance intervention had no short- or long-term effect on PCS or MCS over time (Additional file 1: Table S6 and S7).

**Table 3:** Change of SF-36-based physical component summary (PCS) score and mental component summary (MCS) score [mean and 95% CI] compared to baseline within the per-protocol analysis. Changes were reported as estimated mean differences based on a mixed-model, repeated-measures analysis of variance adjusted for the treatment group, sex, age, and BMI at baseline

Months after weight loss	Change of PCS compared to baseline	Change of MCS compared to baseline
0	5.53 [3.71, 7.34]	3.70 [1.79, 5.60]
12	4.96 [2.84, 7.08]	2.25 [0.09, 4.42]
18	3.82 [1.78, 5.86]	2.06 [-0.08, 4.20]
24	3.11 [1.10, 5.12]	2.00 [-0.28, 4.18]
36	3.18 [1.12, 5.25]	1.73 [-0.89, 4.34]
48	2.95 [0.49, 5.40]	1.79 [-1.00, 4.58]

*PCS* physical component summary, *MCS* mental component summary

### Prediction of long-term modulation of body weight and insulin sensitivity

As previously shown [14], weight loss was associated with substantial changes of ANP receptor balance in adipose tissue. This included a decrease of *NPR-C* mRNA expression as well as a not significant elevation of *NPR-A* mRNA expression during weight loss (Additional file 1: Table S1).

Interestingly, a stepwise linear regression model including adipose *NPR-A* and *NPR-C* mRNA expression after weight loss as well as numerous potential confounders revealed, that lower *NPR-C* expression after weight loss predicted lower body weight regain after 48 months (Table 4). This model was not substantially modified by including a weight loss-induced decrease of 24h urinary metanephrine excretion and adipose β3 adrenoceptor expression after weight loss (Additional file 1: Table S8), while both estimates of weight loss-induced modulation of sympathetic activity were previously shown to predict BMI after 18 months [11]. Comparable evaluation of the adipose ANP system in the context of long-term regulation of body fat percentage confirmed that a lower adipose NPR-C mRNA expression after weight loss predicted a lower increase of body fat percentage between month 0 and month 48 (Additional file 1: Table S9).

**Table 4:** Independent association of NPR-C after weight loss with weight regain at month 48 (ΔBMI_T0T48_). Stepwise multiple linear regression analysis was adjusted for the treatment group, age, sex, and BMI after weight loss

Predictors	Coefficients	Standard error	Standardized *β*	*R*^2^
Adipose *NPR-C* mRNA	0.004	0.002	0.398^*^	0.158*

*NPR* natriuretic receptor

^*^p<0.05

Moreover, lower adipose *NPR-A* mRNA expression after weight loss as well as male sex could be identified as independent predictors of HOMA-IR impairment after weight loss up to 48 months (ΔHOMA-IR_T0T48_) using a comparable linear regression model (Table 5). As ΔBMI_T0T48_ would substantially interact with ΔHOMA-IR_T0T48_, ΔBMI_T0T48_ was also included in this model. In contrast to ΔHOMA-IR_T0T48_, ΔAUC_glucoseT0T48_ was not predicted by the adipose tissue ANP system.

**Table 5:** Independent association of NPR-A after weight loss and male sex with an increase of HOMA-IR at month 48 (ΔHOMA-IR_T0T48_). Stepwise multiple linear regression analysis was adjusted for the treatment group, age, sex, HOMA-IR after weight loss, and ΔBMI_T0T48_. Male sex was defined as 1 and female sex was defined as 2

Predictors	Coefficients	Standard error	Standardized *β*	*R*^2^
Adipose *NPR-A* mRNA	−0.001	3.5×10^-4^	−0.422*	0.361**
Sex	−1.669	0.650	−0.438*	

*NPR* natriuretic receptor

^*^p<0.05

^**^p<0.01

## Discussion

Improvement of health is one of the crucial treatment goals in obesity. This includes modification of numerous metabolic abnormalities as well as numerous factors affecting HRQoL. Unfortunately, the beneficial effects of lifestyle-based weight loss interventions are diminished by frequently observed weight regain [3, 18–20]. Recent data indicate that sustained metabolic improvement and diabetes remission can be achieved by sustained weight maintenance after weight loss in patients with typ 2 diabetes [5]. However, long-term effects up to 48 months as well as the impact on HRQoL have not been addressed in this context so far. Within our trial, we aimed to overcome this knowledge gap by analyzing the impact of a temporary weight maintenance intervention on the beneficial effects of weight loss in the long term regarding obesity, glucose metabolism, and HRQoL. Given the limited resources to perform a lifelong intervention, such a legacy effect would be a potent strategy to improve the long-term outcome of temporary weight loss interventions.

In accordance with previous data [3–5], estimates of insulin sensitivity like HOMA-IR and post-challenge rise of blood glucose were substantially improved by weight loss in our cohort. This beneficial effect on glucose metabolism and insulin sensitivity could be partially preserved for up to 18 or 24 months demonstrating a metabolic long-term benefit of weight loss. Interestingly, compared to baseline body weight reduction partially persists up to 48 months. The divergence between the disappearance of metabolic improvement despite reduced body weight might be caused by the age-related decline of insulin sensitivity in obesity [21]. Similarly, a rise in HbA1c was also reported in participants of the Look AHEAD trial, who could maintain their weight loss for up to 4 years [19].

Both, weight gain and impairment of glucose metabolism could be completely prevented by the 12-month weight maintenance intervention. Unfortunately, these effects were not preserved beyond the end of the maintenance intervention. Thus, a temporary weight maintenance intervention, at least for 12 months, is not eligible to affect long-term weight regain as well as worsening of glucose metabolism. Although we can only speculate about a potential effect of a longer maintenance intervention, current strategies to maintain the metabolic improvement of weight loss should be reconsidered. As continuous lifelong structured support seems not to be feasible and less resource-intensive approaches using technology-supported approaches were not successful [7, 8], repeated short-term interventions after weight loss to inhibit weight regain could represent an alternative concept and should be analyzed in this context. Although we did not focus on type 2 diabetes and diabetes remission, modification of insulin resistance is highly relevant, as improvement of insulin sensitivity is a crucial element in remission of type 2 diabetes. Actually, a combination of weight maintenance and repeated short-term periods of dietary restrictions (if required) was implemented in the DiRECT trial, which demonstrated up to 36% diabetes remission after 24 months [5]. It is therefore tempting to speculate, whether a comparable effect can also be achieved by an initial weight loss intervention combined with repeated dietary short-term restrictions only in those subjects who are characterized by weight regain. Future studies are clearly warranted to address this research question.

Such an approach might be also supported by our HRQoL data. In accordance with previous data [4, 18, 22] a substantial improvement of clinical wellbeing was achieved directly after weight loss. The physical components of HRQoL in particular were enhanced up to 48 months, which confirmed findings of the Look AHEAD study reporting long-term improvement in type 2 diabetics by a weight loss approach [18]. However, the specific effect of a maintenance strategy on HRQoL has not yet been addressed in clinical trials. Somewhat unexpectedly, our data do not necessarily imply an additional benefit of the maintenance intervention, neither in short- nor long-term follow up. This might be due to the fact that improvement of PCS is mainly driven by the degree of weight loss and not by other components [23]. Again, this renders current recommendations to mandatorily combine a weight loss intervention with a maintenance strategy questionable. Although we believe that a successful lifestyle-based maintenance of weight loss over several years might further improve HRQoL, the missing long-term benefit of current strategies makes this assumption debatable and underlines the requirement of alternative approaches.

Given previous data indicating a substantial role of the adipose ANP system on the regulation of fat mass and insulin sensitivity during acute weight loss [14], we further aimed to analyze the impact of this system on long-term body weight regulation and metabolic improvement after weight loss. Importantly, we revealed a lower adipose NPR-C mRNA expression after weight loss in subjects, who will gain less weight after 48 months. Although both sympathetic activity and ANP system are well-known modulators of lipid utilization and weight loss-induced modulation of sympathetic activity predicted weight regain after 18 months [11], the relationship between adipose ANP system and long-term weight regain was not affected by weight loss-induced adaption of sympathetic activity. This indicates an independent effect of the adipose ANP system. The impact of the adipose ANP system on the long-term regulation of obesity was further supported by additional analyses demonstrating that a lower adipose NPR-C mRNA expression after weight loss also predicted a lower increase of body fat percentage after 48 months. Given the increased lipid utilization caused by higher activity of the ANP system (due to lower clearance by adipose NPR-C), this may reflect an underlying mechanism potentially involved in the long-term regulation of fat mass and obesity.

Finally, a higher adipose NPR-A mRNA expression after weight loss was associated with stronger long-term improvement of insulin resistance up to 48 months. As this effect was independent of concomitant BMI changes, the adipose ANP system might be independently relevant for both, long-term regulation of body weight and whole body insulin sensitivity. This substantially extends previous data of our group, demonstrating a substantial role of adipose ANP system in acute regulation of fat mass and insulin sensitivity during weight loss [14]. This may partially help to identify subjects who are prone to weight regain and to develop metabolic re-impairment after weight loss. Moreover, it points out the local ANP system as a promising therapeutic target to improve long-term results of weight loss intervention.

Although the underlying mechanisms are currently unclear, a reduction of adipose tissue clearing receptor C would not only influence systemic ANP levels [15] but also increase local ANP response. Both, the reduction of adipose NPR-C as well as an increase of NPR-A are accompanied by higher local and systemic ANP effects. Thus, the adipose ANP system is also relevant for other insulin target tissue, like skeletal muscle or liver. Such an effect could potentially be mediated by increased secretion of the insulin-sensitizing adiponectin by ANP [24]. Moreover, the ANP system is substantially involved in lipid turnover and ANP-induced lipolysis is attenuated in obesity [25]. Impaired lipolysis negatively impacts lipid turnover which promotes adipose tissue accumulation and consequently obese state [26]. In line with these data, our group and others have already reported a prediction of future weight gain or regain by modulation of genes involved in adipose lipid turnover as well as altered lipolysis [12, 26, 27].

Substantial strengths of the current trial included randomization, the success of the intervention to prevent weight gain until the end of the intervention period, the long duration of the intervention and subsequent repetitive observations, comprehensive phenotyping including adipose tissue biopsies, and the use of well-validated methods and questionnaires.

Nevertheless, the interpretation of our data is also limited by some factors. Several behavioral, social, and environmental factors are known to have a substantial impact on the long-term effects of weight loss interventions [7, 28]. Behavioral factors in particular were not considered in our current analysis. These factors are known to influence body weight trajectory, insulin sensitivity, and QoL. Even if we aimed to standardize the dietary intake and physical activity during the group sessions to sustain weight loss, we cannot completely control dietary behavior. Actually, the length of the long follow-up is an important strength of the trial; however, the concomitant increasing drop-out rate limits the interpretation of our results. The power calculation focused on BMI after 18 months, the primary outcome of the trial, and not on the long-term follow-up of 48 months. Although, the smaller between-group difference of BMI at month 48 compared to month 18 may indicate, that the lack of significance at month 48 is not primarily driven by the lower sample size, confirmatory analyses are required to draw final conclusions. In addition, modulation of cardiac function could also affect weight course in subjects with heart failure. As cardiac function was not assessed within our trial, this represents a limitation. However, severe heart failure represents an exclusion criterion and the presence of mild or subclinical heart failure may have only a minor effect on fluid retention and weight course. Finally, our tissue-specific data are limited as they are based on mRNA expression. The activity of natriuretic receptors is unlikely to be fully reflected by mRNA expression of the respective receptors and may not necessarily represent a functional change. Thus, further studies are clearly required to confirm our data and especially to analyze the question of tissue-specific natriuretic receptor activity in more detail.

## Conclusions

Taken together, our findings support that weight loss has beneficial long-term effects on body weight, insulin sensitivity, and HRQoL, although not all benefits persist up to 48 months after weight loss. Effects on body weight and insulin sensitivity could be only transiently improved by an additional 12-month weight maintenance intervention, unfortunately with no long-term benefit beyond the intervention period. Adaptation of the adipose ANP system to weight loss may be involved in the long-term control of body weight and insulin sensitivity. These data support several efforts to scrutinize the ANP system as a target for future therapeutic approaches in humans.

## Supplementary Information

**Additional file 1: Appendix S1.** Supplementary methods. **Table S1.** Metabolic and anthropometric parameters of the randomized participants before and after weight loss. **Table S2.** BMI [mean and 95% CI] and estimated mean differences within intention to treat analysis. **Table S3.** Frequency of impaired glucose metabolism before (T-3) and after (T0) weight loss. **Table S4.** Change of HOMA-IR and glucose_AUC_ [mean and 95% CI] compared to baseline within per-protocol analysis. **Table S5.** HRQoL of the participants and effects of weight loss. **Table S6.** Physical component summary [PCS] score [mean and 95% CI] and estimated mean differences within per-protocol analysis. **Table S7.** Mental component summary [MCS] score [mean and 95% CI] and estimated mean differences within per-protocol analysis. **Table S8.** Independent association of NPR-C after weight loss with weight regain at month 48 (ΔBMI_T0T48_). **Table S9.** Independent association of NPR-C after weight loss with FM regain at month 48 (ΔFM_T0T48_). **Figure S1.** Flow chart of the randomized controlled trial. f/m indicates female/male. BMI during the randomized controlled trial.

## Data Availability

The datasets generated during and/or analyzed during the current study are available from the corresponding author on reasonable request.

## Abbreviations

ANP: Atrial natriuretic peptide
AUC: Area under the curve
BMI: Body mass index
cGMP: Cyclic guanosine monophosphate
CI: Confidence interval
CV: Coefficient of variance
DGE: German Nutrition Society
FM: Fat mass
glucose_AUC_: Glucose response after oGTT
HDL-cholesterol: High-density lipoprotein cholesterol
HOMA-IR: Homeostatic Model Assessment for insulin resistance
HRQoL: Health-related quality of life
IQR: Interquartile range
LDL-cholesterol: Low-density lipoprotein cholesterol
LOCF: Last observation carried forward
MCS: Mental component summary
NPR: Natriuretic receptor
oGTT: Oral glucose tolerance test
PCS: Physical component summary
SF-36: Short Form 36
