# Supplementary material for: Weight loss-induced improvement of body weight and insulin sensitivity is not amplified by a subsequent 12-month weight maintenance intervention but is predicted by adaption of adipose atrial natriuretic peptide system: 48-month results of a randomized controlled trial
Source: BMC Med. 2022 Jul 28;20:238. doi: 10.1186/s12916-022-02435-9 (PMC9330651; doi:10.1186/s12916-022-02435-9)
Supplement: Supplementary file 1 — Additional file 1: Appendix S1. Supplementary methods. Table S1. Metabolic and anthropometric parameters of the randomized participants before and after weight loss. Table S2. BMI [mean and 95% CI] and estimated mean differences within intention to treat analysis. Table S3. Frequency of impaired glucose metabolism before (T-3) and after (T0) weight loss. Table S4. Change of HOMA-IR and glucoseAUC [mean and 95% CI] compared to baseline within per-protocol analysis. Table S5. HRQoL of the participants and effects of weight loss. Table S6. Physical component summary [PCS] score [mean and 95% CI] and estimated mean differences within per-protocol analysis. Table S7. Mental component summary [MCS] score [mean and 95% CI] and estimated mean differences within per-protocol analysis. Table S8. Independent association of NPR-C after weight loss with weight regain at month 48 (ΔBMIT0T48). Table S9. Independent association of NPR-C after weight loss with FM regain at month 48 (ΔFMT0T48). Figure S1. Flow chart of the randomized controlled trial. f/m indicates female/male. BMI during the randomized controlled trial. [file 12916_2022_2435_MOESM1_ESM.docx]

**Supplemental Material**

**Weight loss induced improvement of body weight and insulin sensitivity were not amplified by a subsequent 12-month weight maintenance intervention but are predicted by adaption of adipose atrial natriuretic peptide system: 48-month results of a randomized controlled trial**

Linna Li, MD^1,2^, Dominik Soll, MD^1,2^, Verena Leupelt, PhD^1,2^, Joachim Spranger, MD^1,2,3^ Knut Mai, MD^1,2,3^

*Appendix S1: Supplementary methods*

*Study design of the pre-trial weight loss phase:*

The protocol of the 12-week weight loss program included three components: caloric restriction, nutritional counseling and physical exercises. Caloric restriction was applied during the weight reduction program in two stages. The first stage based on a replacement of all three meals by a very-low energy diet (Optifast 2^®^, Nestlé HealthCare Nutrition GmbH, Frankfurt am Main, Germany) for eight weeks. This diet supplied 800 kcal per day and was provided by the trial team free of charge. Participants received 35 portions of formula diet for each week (five per day) within the weekly meetings. The participants were advised not to consume any additional food. After eight weeks of very-low energy diet, the diet was switched to an energy-reduced healthy diet to facilitate further weight loss. The diet was composed of a balanced mix with the following distribution of macronutrients: carbohydrates 35-45%, fat 25-35%, and protein 25-30%. In general, daily calorie intake of approximately 1500 kcal was recommended. Based on initial eating protocols, measured energy expenditure and reported physical activity, individual counseling was performed during that period.

This dietary approach was supported by weekly meetings during the entire weight loss period of 12 weeks including dietary counseling for healthy living and recommendations regarding increased physical activity. Within these meetings, nutrition consultants also lead group workshops with practical cooking exercises. Compliance of the diet was supported by providing specific recipes, cooking advice, and instructions for behavior modifications (only three meals per day, at least 4 hours break between the meals, reduced carbohydrate intake at dinner). In the first two months, weight loss and health status were monitored weekly, which included a patient interview and blood pressure measurement. Body weight was measured at once per week. The recommended increase of physical activity was further supported by a supervised 30 min exercise session after each meeting. Participants were also encouraged to attend at least one additional physical exercise course per week. Psychologists also attended the workshops during four dates at week 5, 7, 9 and 11. A physician gave medical advice during one of the meetings as well.

*Study design of the 12 months randomized weight maintenance phase:*

Subjects who lost at least 8% of their body weight during the weight loss phase were considered to be eligible for randomization (n=143). Accordingly, 143 subjects were randomized into an intervention and a control group. A stratified randomization list was used by the study team to allocate participants to groups. Stratification considered gender and body weight at baseline (three BMI strata). Subjects in the control group were no longer involved in any form of counseling. They received an advice leaflet and were asked to return for examination after 12 and 18 months. Subjects in the intervention group received continuous counseling for the next twelve months in gradually decreasing frequency. Weekly group sessions were performed for the first 16 weeks of the 12 months study period. These were comparable to sessions of the weight loss period. Subsequent meetings were performed every two weeks over a period of two months. In the final period meetings occurred once a month until month 12 of the maintenance phase. Thus, at least 36 meetings were offered to every participant of the intervention group during weight maintenance period.

Within the intervention group, the dietary advice was focused on a balanced diet with a comparable distribution of macronutrients as recommended within the final phase of the weight loss intervention. Counseling focused on dietary recommendations advocating the preferential intake of specific foods (like high intake of vegetables, cereals, fat reduced foods, lean meat consumption (lean fish and chicken)). An individual caloric intake was calculated and further adapted to achieve body weight maintenance. Therefore, body weight was measured during every group session. Further weight loss was allowed. In case of body weight gain within this intervention period, a lower energy intake (500 kcal below the calculated energy demand) was recommended.

The supervised physical activity regime was maintained for the first 12
weeks of weight maintenance period. Thereafter participants were encouraged to exercise at least twice a week but without direct supervision. To increase motivation and allow self-monitoring, pedometers were given to the participants and a gym membership was offered. The mentioned psychological support was continued for six additional dates.

*Phenotyping*

The performed phenotyping included anthropometric, hormonal and metabolic evaluation using an oral glucose tolerance test, bioimpedance analysis and assessment of quality of life at every time point. Details of the phenotyping protocol were already described elsewhere (1).

In detail, following a 10-hour overnight fast, body composition was assessed by bioelectric impedance analysis on resting participants using AKERN BIA 101 (SMT medical GmbH & Co. KG, Würzburg, Germany). Afterwards an oral glucose tolerance test (oGTT) with 75 g glucose was performed as described previously (2). Blood samples were taken at 0, 30, 60, 90 and 120 min during oGTT. A *hyperinsulinemic-euglycemic clamp* was also performed (3). In brief, 40 mIU•m^-2^•min^-1^ human insulin (Actrapid®, Novo Nordisk, Bagsvaard, Denmark) and a variable infusion of 10% glucose (Serag Wiessner, Naila, Germany) was used. Capillary glucose concentration was monitored every 5 minutes and was maintained between 4.0 and 4.9 mmol/l via variation of the glucose infusion rate. Blood samples were collected before the clamp and at least two hours after starting the clamp during steady-state conditions. All blood samples were centrifuged, and plasma and serum samples were frozen immediately at -80°C.

Waist circumference was measured three times and the means were calculated. Blood pressure and heart rate were measured likewise, with the participants lying in the horizontal position after 10 min of rest and the means were calculated. Measurement of metanephrine levels was performed in 24-hours urine samples.

Adipose tissue biopsies were taken at T-3 and T0 from different sides to avoid an effect of previous biopsy on mRNA expression at T0. In detail, we obtained abdominal subcutaneous adipose tissue samples (0.5 to 1.0 g) by repeated needle biopsies from the periumbilical region using a 12 G biopty-cut needle (CR Bard GmbH, Karlsruhe, Germany). After anesthetization of skin with 1% lidocaine without epinephrine a skin incision (3-4 mm) was made and biopty-cut needle was used to obtain biopsies. Tissue samples were snap-frozen in liquid nitrogen and stored at -80°C until further analysis.

HRQoL was assessed by Medical Outcomes Study Short Form 36 (SF-36). This questionnaire was completed at each phenotyping visit and represents a self-report HRQoL measure validated across a wide range of clinical and nonclinical populations (4). It provides eight subscale scores: general health perceptions; physical functioning; role limitations due to physical problems; bodily pain; mental health; role limitations due to emotional problems; vitality; and social functioning. In addition, two summary norm-based T-scores are also derived from this questionnaire. First, the physical component summary (PCS) score and second, the mental component summary (MCS) score. The PCS score is derived from general health perceptions; physical functioning; role limitations due to physical problems; bodily pain, and the MCS from mental health; role limitations due to emotional problems; vitality; and social functioning. All scores ranges from 0 to 100. A higher score indicates a better state of HRQoL. For the bodily pain subscale higher scores represent less pain.

*Laboratory analyses*

Capillary blood glucose was measured using the glucose oxidase method (Dr. Müller Super GL, Freital, Germany). Potassium, sodium, calcium, phosphorus, iron, ferritin, serum creatinine, triglycerides, cholesterol, LDL- and HDL-cholesterol, protein, CRP and urea, uric acid, liver enzymes, leucocytes, erythrocytes, thrombocytes, haemoglobin were measured by standard laboratory methods using Cobas ISE direct and c111 Analyzer (Roche Diagnostics, Mannheim, Germany) and Sysmex XE 5000 (Sysmex Deutschland GmbH, Norderstedt, Germany). Serum insulin was measured using fluoroimmunometric assay (AutoDelfia; Perkin Elmer, Rodgau, Germany) (inter-assay CV 2.3 - 3.5 %, intra-assay CV 1.7 - 2.4 %). Urinary metanephrine levels were analyzed by HPLC (Chromsystems Instruments & Chemicals GmbH, Gräfelfing, Germany). Inter-assay CV was 3.8 - 5.4 %.

Total RNA was isolated from about 60-100 mg adipose tissue using TRIzol Reagent (life technologies). Homogenisation was done with the ultra turrax (IKA Labortechnik) in 10 sec cylces for 2-6 times until complete dispersion. cDNA library were prepared from 1 mg total RNA (TruSeq RNA sample Preparation Kit, Illumina San Diego, US). In short, poly-T beads were used to purify poly-A mRNA. The mRNA was fragmented and primed. In a second step double stranded cDNA was synthesized. Adapter 1-12 were used and ligated to the cDNA, to ensure hybridization onto the flow cell. To enrich cDNA fragments containing an adapter sequences on both ends, a short 15 cylce PCR using adapter specific primers was done. Six samples were pooled and loaded onto the flow cell for cluster generation (TruSeq PE Cluster Kit v2-cBot-hs, Illumina San Diego, US). Paired end sequencing was performed on the HiSeq2000 system (TruSeq SBS Kit-Hs 200 cycles, Illumina San Diego, US) running two times 200 cycles. In average about 50*10^6^ reads were obtained. Samples with read depth lower 20*107 and a high number of singletons were filtered out. All quality criteria were fulfilled by 75 adipose tissue pairs (before and after weight loss) and these were used for further analyses. Reads were mapped to the human genome v66 using Tophat (doi:10.1038). Raw counts were extracted using HTSeq and further analyzed using the free statistic software R statistics. For further analysis of the count data, normalization was done using the R package DESeq2 (5).

*References*

1. Mai K, Brachs M, Leupelt V, Jumpertz-von Schwartzenberg R, Maurer L, Gruters-Kieslich A, Ernert A, Bobbert T, Krude H, Spranger J. Effects of a combined dietary, exercise and behavioral intervention and sympathetic system on body weight maintenance after intended weight loss: Results of a randomized controlled trial. Metabolism. 2018;83:60-7.

2. Mai K, Andres J, Bobbert T, Maser-Gluth C, Mohlig M, Bahr V, Pfeiffer AF, Spranger J, Diederich S. Rosiglitazone decreases 11beta-hydroxysteroid dehydrogenase type 1 in subcutaneous adipose tissue. ClinEndocrinol (Oxf). 2007;67(3):419-25.

3. Mai K, Andres J, Biedasek K, Weicht J, Bobbert T, Sabath M, Meinus S, Reinecke F, Mohlig M, Weickert MO, Clemenz M, Pfeiffer AF, Kintscher U, Spuler S, Spranger J. Free fatty acids link metabolism and regulation of the insulin-sensitizing Fibroblast Growth Factor-21. Diabetes. 2009;58(7):1532-8.

4. McHorney CA, Ware JE, Jr., Lu JF, Sherbourne CD. The MOS 36-item Short-Form Health Survey (SF-36): III. Tests of data quality, scaling assumptions, and reliability across diverse patient groups. Med Care. 1994;32(1):40-66.

5. Love MI, Huber W, Anders S. Moderated estimation of fold change and dispersion for RNA-seq data with DESeq2. Genome Biol. 2014;15(550):1-21.

Table S1: Metabolic and anthropometric parameters of the randomized participants before and after weight loss. Results were presented as median and IQR or mean±SD, n represents the number of participants.

| Parameter | n | before weight loss | | after weight loss | | p value |
| --- | --- | --- | --- | --- | --- | --- |
| Females [n (%)] |  | 112 | (78) |  |  |  |
| Postmenopausal females [n (%)] |  | 58 | (51) |  |  |  |
| Age [yr] | 143 | 50.5 | [41.7, 60.8] |  |  |  |
| BMI [kg/m^2^] | 143 | 35.6 | [32.9, 41.0] | 31.3 | [28.7, 36.1] | 3.2*10^-25^ |
| Waist circumference [cm] | 143 | 106.5 | [97.0, 117.0] | 97.0 | [88.0, 107.0] | 1.1*10^-23^ |
| Total cholesterol [mg/dl] | 143 | 203.6 | ±41.8 | 173.8 | ±34.7 | 5.4*10^-21^ |
| HDL-cholesterol [mg/dl] | 143 | 49.3 | [40.6, 61.3] | 47.1 | [39.0, 56.3] | 8.4*10^-10^ |
| LDL-cholesterol [mg/dl] | 143 | 124.8 | ±34.1 | 106.3 | ±30.4 | 3.4*10^-14^ |
| Triacylglycerol [mg/dl] | 143 | 126.0 | [85.0, 169.0] | 84.0 | [64.0, 117.0] | 2.7*10^-17^ |
| HOMA-IR | 142 | 2.2 | [1.4, 3.4] | 1.3 | [0.9, 2.1] | 1.3*10^-15^ |
| AUC_glucose_ [ mg*dl^-1^*min^-1^] | 143 | 277 | [248, 317] | 255 | [231, 301] | 7.5*10^-11^ |
| ISI_Clamp_ [mg•kg^−1^•min^−1^/[mU•l^−1^]] | 139 | 0.06 | [0.04, 0.08] | 0.08 | [0.07, 0.10] | 6.2*10^-19^ |
| Systolic blood pressure [mmHg] | 138 | 127.7 | [120.0, 138.1] | 119.0 | [111.3, 126.4] | 1.4*10^-14^ |
| Diastolic blood pressure [mmHg] | 138 | 80.5 | [73.0, 87.3] | 73.0 | [68.3, 81.4] | 1.6*10^-9^ |
| Adipose NPR-A mRNA expression | 75 | 4560 | [4052, 5202] | 4786 | [4370, 5386] | 0.051 |
| Adipose NPR-C mRNA expression | 75 | 919 | [690, 1330] | 459 | [279, 759] | 1.4*10^-10^ |

BMI, body mass index; HDL-cholesterol, high density lipoprotein cholesterol; LDL-cholesterol, low density lipoprotein cholesterol; HOMA-IR, Homeostatic Model Assessment for insulin resistance; glucoseAUC, Area under the curve of glucose response after oGTT; ISI_Clamp_, Insulin sensitivity index assessed by hyperinsulinemic euglycemic clamp; NPR, natriuretic receptor.

Table S2: BMI [mean and 95% CI] and estimated mean differences within intention to treat analysis.

Intervention effects reported as estimated marginal means and estimated mean differences (intervention minus control) based on mixed-model, repeated-measures analysis of variance adjusted for treatment group, gender, age and BMI at baseline.

| Months after  weight loss | Intervention  group | Control  group | Group difference |
| --- | --- | --- | --- |
| 0 | 32.27 [31.93, 32.62] | 32.66 [32.31, 33.01] | -0.39 [-0.06, 0.84] |
| 12 | 32.34 [31.63, 33.05]** | 33.64 [32.92, 34.36] | -1.30 [-2.29, -0.31] |
| 18 | 33.51 [32.79, 34.23] | 34.20 [33.48, 34.93] | -0.70 [-1.70, 0.31] |
| 24 | 33.76 [33.02, 34.50] | 34.38 [33.63, 35.13] | -0.62 [-1.65, 0.41] |
| 36 | 34.01 [33.30, 34.71] | 34.55 [33.83, 35.26] | -0.54 [-1.53, 0.44] |
| 48 | 34.13 [33.41, 34.84] | 34.65 [33.94, 35.37] | -0.53 [-1.52, 0.46] |

* p=0.01 vs. control group

Table S3: Frequency of impaired glucose metabolism before (T-3) and after (T0) weight loss.

| Glucose metabolism | before weight loss | after weight loss |
| --- | --- | --- |
| Normal glucose tolerance [n [%]] | 87 [60.8] | 98 [68.5] |
| Diabetes mellitus, IGT or IFG [n [%]] | 56 [39.2] | 45 [31.5] |

Pairwise McNemar´s test: p=0.071; IFG, impaired fasting glucose; IGT, impaired glucose tolerance

Table S4: Change of HOMA-IR and glucose_AUC_ [mean and 95% CI] compared to baseline within per-protocol analysis.

Changes were reported as estimated mean differences based on mixed-model, repeated-measures analysis of variance adjusted for treatment group, gender, age and BMI at baseline.

| Months after  weight loss | Change of HOMA-IR compared to baseline | Change of glucose_AUC_ compared to baseline [mg*dl^-1^*min^-1^] |
| --- | --- | --- |
| 0 | -1.60 [-1.99, -1.21] | -27.51 [-36.81, -18.22] |
| 12 | -1.31 [-1.71, -0.92] | -11.59 [-21.64, -1.55] |
| 18 | -0.91 [-1.35, -0.47] | 0.61 [-9.57, 10.79] |
| 24 | -0.97 [-1.37, -0.56] | 11.30 [0.60, 22.00] |
| 36 | -0.49 [-1.00, 0.02] | 28.61 [16.86, 40.36] |
| 48 | -0.14 [-0.64, 0.36] | 35.40 [22.75, 48.05] |

HOMA-IR, Homeostatic Model Assessment for insulin resistance; glucoseAUC, Area under the curve of glucose response after oGTT

Table S5: HRQoL of the participants and effects of weight loss.

Parameters of HRQoL of the randomized participants before and after weight loss. Results were presented as median and IQR, n represents the number of participants.

| Parameter | n | before weight loss | | n | after weight loss | | p value |
| --- | --- | --- | --- | --- | --- | --- | --- |
| physical functioning | 142 | 70.0 | [50.0, 86.0] | 142 | 85.0 | [75.0, 95.0] | 2.5*10^-16^ |
| role limitations due to physical problems | 134 | 100.0 | [25.0, 100.0] | 141 | 100.0 | [75.0, 100.0] | 3.8*10^-5^ |
| bodily pain | 139 | 62.0 | [41.0, 100.0] | 141 | 74.0 | [52.0, 100.0] | 5.1*10^-6^ |
| general health perceptions | 132 | 63.5 | [47.8, 77.0] | 138 | 72.0 | [57.0, 82.0] | 1.5*10^-7^ |
| vitality | 133 | 50.0 | [35.0, 60.0] | 139 | 65.0 | [50.0, 75.0] | 6.0*10^-16^ |
| social functioning | 142 | 81.3 | [62.5, 100.0] | 142 | 100.0 | [75.0, 100.0] | 8.3*10^-5^ |
| role limitations due to emotional problems | 134 | 100.0 | [33.0, 100.0] | 140 | 100.0 | [100.0, 100.0] | 1.6*10^-3^ |
| mental health | 133 | 68.0 | [52.0, 80.0] | 139 | 76.0 | [64.0, 88.0] | 1.0*10^-7^ |
| PCS score | 125 | 46.0 | [36.0, 53.4] | 137 | 51.7 | [45.1, 55.8] | 7.8*10^-9^ |
| MCS score | 125 | 49.6 | [39.4, 54.8] | 137 | 53.4 | [47.9, 57.3] | 7.4*10^-5^ |

HRQoL, health related quality of life; PCS, physical component summary; MCS, mental component summary

Table S6: Physical component summary [PCS] score [mean and 95% CI] and estimated mean differences within per-protocol analysis.

Intervention effects reported as estimated marginal means and estimated mean differences [intervention minus control] based on mixed-model, repeated- measures analysis of variance adjusted for treatment group, gender, age and BMI at baseline.

| Months after  weight loss | Intervention  group | Control  group | Group difference |
| --- | --- | --- | --- |
| 0 | 49.85 [47.83, 51.87] | 50.66 [48.59, 52.74] | -0.81 [-3.44, 1.82] |
| 12 | 49.57 [47.11, 52.03] | 49.36 [46.77, 51.95] | 0.21 [-3.16, 3.57] |
| 18 | 48.32 [45.87, 50.76] | 48.82 [46.24, 51.40] | -0.50 [-3.85, 2.85] |
| 24 | 48.51 [45.83, 51.19] | 47.08 [44.27, 49.89] | 1.43 [-2.27, 5.14] |
| 36 | 46.42 [43.73, 49.10] | 49.58 [46.59, 52.58] | -3.17 [-7.01, 0.67] |
| 48 | 47.25 [44.33, 50.17] | 47.81 [44.50, 51.12] | -0.56 [-4.82, 3.70] |

Table S7:  Mental component summary [MCS] score [mean and 95% CI] and estimated mean differences within per-protocol analysis.

Intervention effects reported as estimated marginal means and estimated mean differences [intervention minus control] based on mixed-model, repeated- measures analysis of variance adjusted for treatment group, gender, age and BMI at baseline.

| Months after  weight loss | Intervention  group | Control  group | Group difference |
| --- | --- | --- | --- |
| 0 | 49.76 [47.14, 52.39] | 49.48 [46.79, 52.17] | 0.29 [-3.17, 3.74] |
| 12 | 47.59 [44.74, 50.44] | 48.98 [45.98, 51.98] | -1.40 [-5.27, 2.48] |
| 18 | 46.28 [43.35, 49.21] | 48.79 [45.68, 51.89] | -2.51 [-6.53, 1.52] |
| 24 | 47.24 [44.13, 50.34] | 48.50 [45.24, 51.76] | -1.26 [-5.54, 3.01] |
| 36 | 47.95 [44.46, 51.44] | 47.14 [43.12, 51.16] | 0.81 [-4.34, 5.97] |
| 48 | 47.40 [43.72, 51.07] | 47.75 [43.62, 51.89] | -0.36 [-5.71, 5.00] |

Table S8: Independent association of NPR-C after weight loss with weight regain at month 48 (ΔBMI_T0T48_).

Stepwise multiple linear regression analysis was adjusted for treatment group, age, sex, BMI after weight loss as well as weight loss induced decrease of 24h urinary metanephrine excretion and adipose β3 adrenoceptor expression after weight loss.

| Predictors | Coefficients | Standard error | Standardized β | R^2^ |
| --- | --- | --- | --- | --- |
| Adipose *NPR-C* mRNA | 0.004 | 0.002 | 0.445^*^ | 0.198* |

*p<0.05

NPR, natriuretic receptor.

Table S9: Independent association of NPR-C after weight loss with FM regain at month 48 (ΔFM_T0T48_).

Stepwise multiple linear regression analysis was adjusted for treatment group, age, sex and FM percentage after weight loss.

| Predictors | Coefficients | Standard error | Standardized β |  | R^2^ |
| --- | --- | --- | --- | --- | --- |
| Adipose *NPR-C* mRNA | 0.008 | 0.004 | 0.391* |  | 0.338** |
| FM after weight loss (T0) | -0.335 | 0.105 | -0.568** |  |  |

*p<0.05; **p<0.01

NPR, natriuretic receptor.

**Supplemental figures** **legends**

Figure S1. Flow chart of the randomized controlled trial. f/m indicates female/male.

Figure S1

Assessed for eligibility (n=223)

## Enrollment

Excluded (n= 67)

♦  Not meeting inclusion criteria (n=67)

Inclusion in 12-week weight reduction program (n= 156)

Drop out (n = 13 (8.3%))

♦ Lost interest (n=5)

♦ Unable to achieve weight loss ≥ 8% (n=6)

♦ Unable to attend group sessions (n=2)

Analyzed after 12 months (n=58)

(f 45; m 13)

♦ Not available for follow-up (unable to be contacted) (n=13)

Analyzed after 18 months (n=51)

(f 39; m 12)

♦ Not available for follow-up (unable to be contacted) (n=7)

Analyzed after 24 months (n=40)

(f 30; m 10)

♦ Not available for follow-up (unable to be contacted) (n=11)

Analyzed after 36 months (n=25)

(f 15; m 10)

♦ Not available for follow-up (unable to be contacted) (n=15)

Analyzed after 48 months (n=22)

(f 15; m 7)

♦ Not available for follow-up (unable to be contacted) (n=3)

## Follow up

Analyzed after 48 months (n=33)

(f 23; m 10)

♦ Not available for follow-up (unable to be contacted) (n=6)

Analyzed after 36 months (n=39)

(f 29; m 10)

♦ Not available for follow-up (unable to be contacted) (n=6)

Analyzed after 24 months (n=45)

(f 34; m 11)

♦ Not available for follow-up (unable to be contacted) (n=16)

Analyzed after 18 months (n=61)

(f 48; m 13)

♦ Not available for follow-up (unable to be contacted) (n=3)

Analyzed after 12 months (n=64)

(f 50; m 14)

♦ Not available for follow-up (unable to be contacted) (n=8)

## Allocation

Allocated to control group (n=71)

(f 56; m 15)

♦ Received allocated intervention (n=71)

## 12 months randomized intervention

Allocated to intervention group (n=72)

(f 56; m 16)

♦ Received allocated intervention (n=72)

Randomized (n=143)
